# Multiple Sclerosis Presenting with Facial Twitching (Myokymia and Hemifacial Spasms)

**DOI:** 10.1155/2017/7180560

**Published:** 2017-09-17

**Authors:** Risha Hertz, James Espinosa, Alan Lucerna, Doug Stranges

**Affiliations:** ^1^University of Pennsylvania Health System, Penn Medicine, Gibbsboro, NJ, USA; ^2^Department of Emergency Medicine, Rowan University SOM, Stratford, NJ, USA

## Abstract

Multiple sclerosis (MS) is a chronic inflammatory demyelinating disease of the central nervous system. The etiology is insufficiently understood. Autoimmune, genetic, viral, and environmental factors have been hypothesized. MS is twice as common in women as in men between the ages of 20 and 50 years. There is no known cure for MS. Current medical treatment helps to prevent new attacks and improve function after an attack. MS is diagnosed by physical examination, diagnostic imaging, and examination of cerebral spinal fluid. The most common physical signs and symptoms of MS include constitutional symptoms, muscle weakness, motor and autonomic spinal cord symptoms, paresthesias, and vision changes. Here we present a case of MS diagnosed in a 33-year-old male with facial myokymia of left eyelid, which progressed to left hemifacial spasm. This is an unusual presentation for multiple sclerosis. An awareness of this presentation not only may lead to an earlier diagnosis in some patients but can be a sign of relapse in patients with established multiple sclerosis.

## 1. Introduction

Multiple sclerosis (MS) is a chronic inflammatory demyelinating disease of the central nervous system. The etiology is insufficiently understood. Most common causes are thought to be related to autoimmune, genetic, viral, and environmental factors. MS is twice as common in women as in men between the ages of 20–50 years. There is no known cure for MS. Current medical treatment helps to prevent new attacks and improve function after an attack. MS is diagnosed by physical examination, diagnostic testing including imaging, and examination of cerebral spinal fluid. The most common physical signs and symptoms of MS include constitutional symptoms, muscle weakness, motor and autonomic spinal cord symptoms, paresthesias, and vision changes.

## 2. The Case

A 33-year-old male presents with several weeks of what he described as spasms of his left eyelid which progressed to episodes of twitching of his left eye and left cheek. A comprehensive review of systems was negative. He denied history of tobacco, drug, or alcohol abuse. His past medical history included gastroesophageal reflux disease and osteogenesis imperfecta. His past surgical history and family history were noncontributory. He did not take any prescribed, herbal, or over the counter medications. He drank one to two cups of coffee daily. His vital signs were within normal limits. His vital signs were normal on presentation. He was orientated to person, place, and time and well-developed, well nourished, and in no distress. Spasms were noted of his left eyelid with several brief episodes of left hemifacial spasm. His physical exam was otherwise within normal limits.

Diagnostic testing included magnetic resonance imaging (MRI) of the brain, magnetic resonance angiogram (MRA) of the head and neck, complete blood count with differential, comprehensive metabolic panel, magnesium, phosphorus, thyroid stimulating hormone, free thyroxine, lipid panel, and an erythrocyte sedimentation rate (ESR). The MRA showed no evidence of a hemodynamically significant intracranial stenosis, occlusion, or aneurysm. An MRI of the brain was performed at 3T with the following sequences: 3D sagittal T1, axial reformat from 3D sagittal FLAIR, axial proton density-weighted image and FLAIR, axial diffusion-tensor imaging trace and ADC, and axial reformats from post-contrast sagittal 3D T1-weighted image. The MRI showed T2-weighted lesions. There was a round focus of hyperintense signal within the left anterior hemisphere measuring up to six millimeters. There was a hyperintense signal within the right parasagittal pons. There was a dominant focus of hyperintense signal lateral to the right atrium extending to the right posterior medial temporal lobe measuring up to 18 millimeters. There was a round focus of hyperintense signal within the left inferior frontal subcortical white matter. Additional foci of hyperintense signal were seen within the right and left periatrial white matter as well as within the bilateral corona radiata and centrum semiovale. The findings were interpreted as consistent with multiple sclerosis. His blood work was unremarkable except for a borderline low Vitamin D level (30 ng/ml), a slightly elevated ESR (40 mm/h).

The patient was referred to Neurology. The patient had multiple T2 lesions in at least 2 of 4 MS-typical regions of the CNS in addition to the myokymia. A diagnosis of multiple sclerosis was made. It was felt by Neurology that the location of the lesions would be consistent with his symptoms.

He was started on Copaxone (glatiramer acetate) as well as Vitamin D supplementation.

The patient has been followed by Neurology and has been doing well with the current treatment in the six months following the diagnosis. He was not treated with botulinum toxin. He was not treated with corticosteroids.

## 3. Discussion

The patient's presentation with isolated eyelid twitching is consistent with myokymia. Myokymia is defined as muscle twitching of the nature of undulating vermicular muscle movements under the skin without contractures that move the face [1]. It may progress to facial contractures, which can be sustained.

Myokymia, with or without progression to facial intermittent spasm, and with or without sustained contractures, is said in the literature to be an uncommon presentation of multiple sclerosis. It can sometimes be seen during a relapse [2]. The etiology is unclear but hyperexcitability of the facial neurons secondary to demyelination of corticospinal tract fibers is proposed [3].

Spastic contractures of facial muscles associated with multiple sclerosis can be nonsustained. When nonsustained, such contractures have been called hemifacial spasm (HFS) in the literature. Sustained contractures have been called spastic paretic hemifacial contracture (SPHC).

Electromyography (EMG) studies suggest that nonsustained contractures (HFS), spastic contractures (SPHC), and facial myokymia are related. The difference in the EMG findings appears to be whether individual muscles or groups of muscles are involved (myokymia) and whether the firing is nonsustained (HFS) or sustained (SPHC) [2–4]. Facial myokymia and facial spasms in the context of multiple sclerosis have been treated with botulinum toxin injections [5].

In a 1994 case series of twelve patients with MS and facial myokymia, Jacobs et al. observed consistent MRI findings in the postnuclear, postgenu portion of the facial nerve that resolved when myokymia stopped [6].

MRI is utilized today as a marker in treatment response. Wattjes et al. state that “the detection of disease activity (or its absence) by MRI in a patient that receives an immunomodulatory treatment represents a measure of treatment response” [7]. In a patient undergoing immunomodulatory or immunosuppressive treatment for relapsing-remitting MS, myokymia might correspond to a MS relapse, indicating disease activity despite actual therapy, so optimizing therapy might be considered. It could also be suggested that myokymia may prompt consideration of MS as a new diagnosis, as in this case.

Facial myokymia, HFS, and SPHC are not pathomneumonic for multiple sclerosis. Transient facial myokymia can be due to such benign causes as fatigue, excessive caffeine, anxiety, eye muscle fatigue, and mild magnesium deficiency. Stress and dehydration can be associated.

Facial myokymia has also been described in the recovery phase of Guillain-Barre syndrome.

Progressive SPHC has been seen in brainstem tumors [2, 3]. HFS has been seen in vertebral aneurysm and with enlarged vertebrobasilar vessels. It has been seen with enlarged anomalous vessels that compress the seventh nerve at the level of the brainstem, as well as with cholesteatomas and acoustic neuromas [4].

## 4. Conclusions

Here we present an unusual presentation of multiple sclerosis diagnosed in a 33-year-old male with facial myokymia of left eyelid, which progressed to left hemifacial spasm. Myokymia, with or without progression to facial intermittent spasm, and with or without sustained contractures, is said in the literature to be an uncommon presentation of multiple sclerosis but could be suggestive of a relapse [2]. The etiology is unclear but hyperexcitability of the facial neurons secondary to demyelination of corticospinal tract fibers is proposed [3]. Facial myokymia, HFS, and SPHC are not pathomneumonic for multiple sclerosis.

An awareness of this presentation may lead to a diagnosis of MS in some patients and can be a sign of relapse in patients with established multiple sclerosis.
